# Efficacy and safety of vildagliptin, Saxagliptin or Sitagliptin as add-on therapy in Chinese patients with type 2 diabetes inadequately controlled with dual combination of traditional oral hypoglycemic agents

**DOI:** 10.1186/1758-5996-6-69

**Published:** 2014-05-31

**Authors:** Chun-Jun Li, Xiao-Juan Liu, Lian Bai, Qian Yu, Qiu-Mei Zhang, Pei Yu, De-Min Yu

**Affiliations:** 1 Department of Endocrinology, 2011 Collaborative Innovation Center of Tianjin for Medical Epigenetics, Key Laboratory of Hormone and Development (Ministry of Health), Metabolic Disease Hospital & Tianjin Institute of Endocrinology, Tianjin Medical University, Tianjin, China

**Keywords:** Type 2 diabetes mellitus, Glycemic control, DPP-4 inhibitors, OHAs

## Abstract

**Background:**

The oral DPP-4 inhibitors are new incretin-based therapies for treatment of type 2 diabetes. To assess the efficacy and safety of three DPP-4 inhibitors (Saxagliptin, Sitagliptin and Vildagliptin) as add-on therapy to dual combination of traditional oral hypoglycemic agents in Chinese type 2 diabetes patients.

**Methods:**

In this 24-week, randomized, open-label, parallel clinical trial, we enrolled inadequately controlled (glycosylated haemoglobin A1c [HbA1c] ≥7.5% to ≤10%) patients with type 2 diabetes, who were treated by dual combination of metformin and another traditional oral hypoglycemic agent (glimepiride, acarbose or pioglitazone). 207 patients had been randomized to add-on 5 mg saxagliptin group or 100 mg sitagliptin once daily group, or 50 mg vildagliptin twice daily group for 24 weeks. HbA1c, fasting and postprandial blood glucose (FBG and P2hBG), body weight, body mass index (BMI), episodes of hypoglycemia and adverse events were evaluated.

**Result:**

After 24 weeks, HbA1c, FBG, and P2hBG of each group were significantly decreased. (saxagliptin vs vildagliptin vs sitagliptin: HbA1c: -1.2% vs -1.3% vs -1.1%; FBG: -1.8 mmol/l vs -2.4 mmol/l vs -1.5 mmol/l; P2hBG: -3.4 mmol/l vs -3.7 mmol/l vs -3.2 mmol/l). The changes of HbA1c and P2hBG among the three groups had no significance. However, vildagliptin-added group showed the greatest reduction (p < 0.001), while, sitagliptin-added group showed the lowest reduction (p < 0.001) in terms of FPG changes. Proportions of patients achieving HbA1c < 7% at the end were similar in three groups (saxagliptin 59%, vildagliptin 65%, sitagliptin 59%). Mild hypoglycemia was commonly reported among the three groups (saxagliptin 6%, vildagliptin 2%, sitagliptin 3%). No significant between-group difference was shown in other AEs.

**Conclusion:**

The three gliptins showed almost similar glycemic control and incidence of adverse events. However, for FBG control, saxagliptin demonstrated superiority to sitagliptin, while, inferiority to vildagliptin.

## Background

Type 2 diabetes mellitus (T2DM) is a complex disease mainly caused by impaired beta cell function and insulin resistance. In China, a total diabetes prevalence of 9.7% (92.4 million adults) was reported by the China National Diabetes and Metabolic Disorders Study in 2007–2008, while the prevalence of pre-diabetes was estimated to be 15.5% (148 million adults) [1]. Moreover, the prevalence of T2DM in Asia is expected to increase over the next 20 years due to a sedentary lifestyle combined with the increase in obesity and overweight resulting from economic development, and the changes in diet [2]. The increasing prevalence of type 2 diabetes and inadequate control of blood glucose in patients correlates with higher risk for cataracts, retinopathy, neuropathy, and other diabetic microvascular complications [3,4]. There is also a strong correlation between mean glycated hemoglobin (HbA1c) levels over time and the development and progression of diabetic complications. Both the American Diabetes Association and the Chinese Diabetes Society advocate a HbA1c goal of <7.0% for individuals with T2DM [5,6], although adequate glycemic control is difficult to achieve in China [1,7]. Therefore, early and long-term control of type 2 diabetes is required.

According to Chinese guidelines for T2DM treatment, metformin is recommended when diet and lifestyle interventions alone are unable to maintain blood glucose control at target levels [8,9]. Failure of monotherapy over time suggests the need for combination therapy to achieve or maintain glycemic goals [10]. Several oral therapies are approved for use in combination with metformin; however, they are not always effective and are associated with side effects [11]. Sulfonylureas are associated with hypoglycemia and weight gain; thiazolidinediones are associated with weight gain, fluid retention, congestive heart failure, and fractures; and α- glucosidase inhibitors are associated with abdominal discomfort, increased intestinal gas, and diarrhea [11]. Given these considerations, there remains a substantial unmet need for an agent that could improve β-cell function, improve glycaemic control, and have less adverse effects.

Dipeptidyl peptidase-4 (DPP-4) inhibitors are a new class of oral anti-diabetic agents that increase circulating concentrations of the glucagon-like peptide-1 (GLP-1) [12]. GLP-1 released after meals but degraded by dipeptidyl peptidase-4 (DPP-4) rapidly. The DPP-4 inhibitors block the rapid inactivation of GLP-1 and improve glycaemic control [13]. It has been indicated that dipeptidyl peptidase-4 inhibitors (DPP4i) are superior to traditional oral hypoglycemic agents in terms of the efficacy and tolerability [14-17]. Such priority can be also seen from the rising status in the authoritative guideline for preventing and treating diabetes. In the 2009 position statement of the American Diabetes Association (ADA) and the European Association for the Study of Diabetes (EASD) for the management of hyperglycemia in type 2 diabetes, DPP4i were considered as less well-validated therapies and not recommended in the main therapy steps [18]. However, the 2012 statement of the ADA and EASD included DPP4i as second-option medication when metformin fails, despite there was no specific preference compared with other oral agents (sulfonylurea and thiazolidinedione) in the same position [19].

Several DPP-4 inhibitors have been produced, including vildagliptin, sitagliptin, and saxagliptin [20].

For monotherapy in Asian countries, all of the three agents are demonstrated to be effective and safe comparing with placebo [21-23]. When it comes to combination therapy, the three agents demonstrated great glycemic control and tolerance in Asian patients with T2DM who had inadequate glycemic control with metformin or glimepiride [24-28]. So far, no trial has been conducted head-to-head comparing the three DPP-4 inhibitors in Asian. The current clinical trial was designed as a prospective study to investigate the efficacy and safety of vildagliptin, saxagliptin, or sitagliptin added to the existing therapy in Chinese type 2 diabetes patients, who had inadequate glycemic control with conventional oral hypoglycemic agents.

## Methods

### Study design

This was a 24-week, randomized, open-label, parallel clinic study in patients with T2DM who had inadequate glycaemic control with dual combination of metformin and other traditional oral hypoglycemic agents (glimepiride, acarbose and pioglitazone). Patients were screened for the eligibility at Visit 1 (week -12) and were randomized (1 : 1: 1) at Visit 2 (week 0, baseline) to receive saxagliptin 5 mg qd, vildagliptin 50 mg bid, or sitagliptin 100 mg qd for 24 weeks. The randomization was stratified on the basis of the patient’s background treatment into metformin with glimepiride, or acarbose, or pioglitazone combination therapy. During the 24-week treatment period, patients were required to visit the outpatient once every two weeks, and telephone contact every week. The dosage of the background oral hypoglycemic agents was adjusted through the whole treatment period if the hypoglycemia occurred, based on the value of blood glucose and physicians’ experience.

### Study population

Patients were recruited from the Metabolic Disease Hospital of Tianjin Medical University between Jan 2012 and Jan 2013. All subjects provided written informed consent and confirmed their willingness to perform glucose self-monitoring. This study design was approved by the local ethics committee review board and was conducted using Good Clinical Practice in accordance with the Declaration of Helsinki.

Patients with T2DM (aged 18–70) who were inadequately controlled by dual combination of traditional oral hypoglycemic agents with HbA1c of 7.5–10.0% and body mass index (BMI) of 22.5–30 kg/m2 were eligible for enrollment. Patients were required to have been treated with metformin and another oral hypoglycemic agent (glimepiride, acarbose, or pioglitazone) for at least 12 weeks and be on a stable recommended dose.

Patients were excluded if they had a history of type 1 diabetes mellitus or diabetes due to pancreatic injury or secondary forms of diabetes, any acute metabolic diabetic complications such as ketoacidosis or hyperosmolar state (coma) within past 6 months, myocardial infarction, unstable angina or coronary artery bypass surgery within past 6 months. Patients with congestive heart failure, liver disease such as cirrhosis or chronic active hepatitis or with any of the following laboratory abnormalities at Visit 1 were also excluded: alanine aminotransferase (ALT) or aspartate aminotransferase (AST) > 2 times the upper limit of normal (ULN), total bilirubin > 2 times ULN, serum creatinine levels [men: ≥ 1.5 mg/dl (132 μmol/l); women: ≥ 1.4 mg/dl (123 μmol/l)] or thyroid-stimulating hormone beyond the normal range, fasting triglycerides > 500 mg/dl (5.6 mmol/l).

### Study assessments

Assessments included HbA1c, FBG and P2hBG at baseline and the endpoint of 24 weeks.

Adverse events (AEs) were recorded and assessed for their severity and the potential relationship to the study drug. Patients were educated how to recognize the signs and symptoms of hypoglycemia and asked to test glucose whenever they experienced such events and to record the results if possible. Hypoglycaemia was defined as the presence of symptoms suggestive of hypoglycaemia, confirmed by self-monitored blood glucose < 56 mg/dl (3.1 mmol/l). Severe hypoglycaemia was defined as an episode requiring assistance of another party. All assessments were performed in a central laboratory with standardized and validated procedures according to Good Laboratory Practice.

### Statistical analysis

Results are described as mean ± standard deviation or n and%. 2-tailed paired *t*-test was used for comparison of pre- and post-treatment values within group. One-way univariate analysis of variance (ANOVA) and polynomial was used to compare the differences in clinical characteristics among the three groups at baseline and after treatment assessed for significance using for the discrete or continuous data. For analysis of differences in the frequency distributions, the chi-square test and Fisher’s exact test were used. Statistical analyses were performed using SPSS windows version 19.0. P value < 0.05 was considered to be statistically significant.

## Results

### Patients

**Figure 1:**
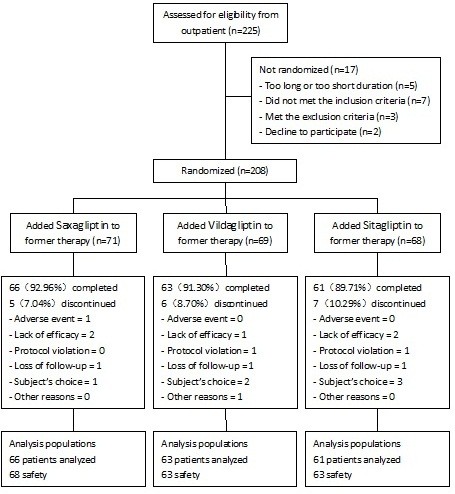
Specific information of study population, allocation and withdrawal in present clinical study.

Between Jan 2012 and Jan 2013, A total of 208 patients were randomized, and 190 patients comprised the full analysis set. 5 of 71 subjects withdrew from saxagliptin, 6 of 69 from vildagliptin and 7 of 68 from sitagliptin treatment. The withdrawal rates were not significantly different between the groups. The disposition of patients and the reasons for withdraw were summarized in Figure 1. According to treatment group, 66 patients were analyzed in saxagliptin group, 63 in vildagliptin group, and 61 in sitagliptin group. The patients’ demographic and baseline characteristics are described in Table 1. No significant difference was observed in baseline demographics and metabolic characteristics among the three groups after randomization.

**Table 1:** Demographic and baseline characteristics

	**Total n = 190**	**Saxagliptin n = 66**	**Vildagliptin n = 63**	**Sitagliptin n = 61**	**p value**
Age (years), mean ± SD	46.6 ± 9.0	46.5 ± 10.7	44.8 ± 8.5	48.6 ± 11.3	0.90
Range	23 ~ 72	23 ~ 72	25 ~ 63	29 ~ 70	-
Age (years), n (%)					
<50 years	53(28%)	18(27%)	18(29%)	17(27%)	0.99
50 ~ 65 years	90(47%)	31(47%)	29(46%)	30(50%)	0.94
>65 years	47(25%)	17(26%)	16(25%)	14(23%)	0.93
Gender, n (%)					
Male	109(57%)	39(59%)	37(59%)	33(54%)	0.82
Female	81(43%)	27(41%)	26(41%)	28(46%)	0.82
Duration of diabetes (years), n (%)					
<1 year	61(32%)	20(30%)	24(38%)	17(28%)	0.44
1 ~ 5 years	69(36%)	24(37%)	21(33%)	24(40%)	0.79
>5 years	60(32%)	22(33%)	18(29%)	20(32%)	0.82
Body weight (kg), mean ± SD	74.4 ± 11.1	77.2 ± 15.1	72.7 ± 11.3	73.2 ± 10.9	0.89
BMI (kg/m2), mean ± SD	26.3 ± 2.8	26.9 ± 3.1	25.3 ± 2.8	26.6 ± 3.3	0.80
HbA1c (%), mean ± SD	8.72 ± 1.01	8.86 ± 1.13	8.75 ± 1.15	8.54 ± 1.19	0.94
FBG (mmol/l), mean ± SD	8.46 ± 1.61	8.36 ± 2.04	8.79 ± 1.80	8.22 ± 1.77	0.93
P2hBG(mmol/l), mean ± SD	11.58 ± 2.49	11.77 ± 3.07	11.98 ± 2.46	10.98 ± 2.93	0.90
Therapy, n (%)					
Metformin with glimepiride	77(41%)	26(40%)	26(41%)	25(41%)	0.97
Metformin with acarbose	62(33%)	22(33%)	20(32%)	20(33%)	0.98
Metformin with pioglitazone	51(26%)	18(27%)	17(27%)	16(26%)	0.99

*Abbreviations*: *BMI* body mass index; *FBG* fasting blood glucose; *P2hBG* postprandial 2 hours’ blood glucose.

Data are presented as mean ± standard deviation, as well as, n and%.

### Glycaemic control

**Figure 2:**
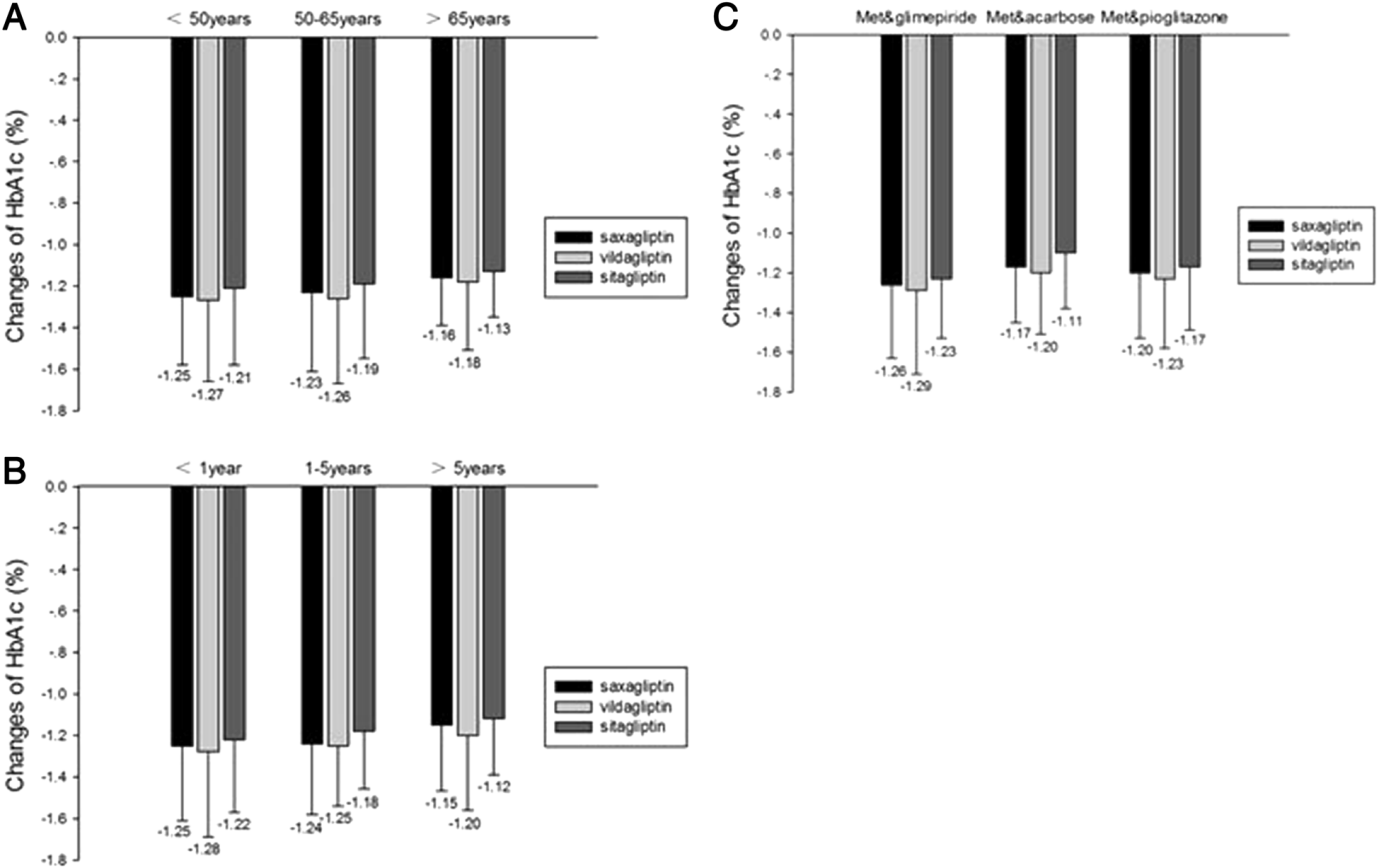
**Changes of HbA1c from baseline to the end in different parts.** The black bar indicates the HbA1c of saxagliptin-added group, the white bar indicates that of vildagliptin-added group and the gray bar indicates that of sitagliptin-added group. Bars represent the differences of the mean HbA1c from baseline to the end. Picture **A** shows the changes in three age stages (<50 years, 50-64years, and >65 years). Picture **B** shows the changes in three duration stages (<1 year, 1-5years, and >5 years). Picture **C** shows the changes in three different background therapeutic regimens (metformin with glimepiride, metformin with acarbose, metformin with pioglitazone). Repeated measured ANOVA test and polynomial were used for statistical analysis.

The changes of HbA1c, FBG, and P2hBG from baseline to end were performed in Table 2. After 24 weeks, HbA1c, FBG, and P2hBG of each group were significantly decreased. Although, the changes of HbA1c and P2hBG among the three groups were not significant differences. Saxagliptin-added group (-1.8 ± 0.08) was greater than sitagliptin-added group (-1.5 ± 0.05, p = 0.038), but less than vildagliptin-added group in FBG reduction (-2.4 ± 0.06, p = 0.003).The patients were divided into different parts to find out whether the three DPP-4 inhibitors had special effects on diverse ages, duration, or background drugs. The results were displayed in Figure 2. The reductions in HbA1c among the three DPP-4i-added groups of all the subgroups had no significant difference.

**Table 2:** **Changes of variables related with HbA**_**1c**_**, FBG, and P2hBG after 24-week treatment**

	**Baseline (mean ± s.d)**	**24 weeks (mean ± s.d)**	**Mean changes from baseline (95% CI)**	**★Difference in mean change (95% CI)**	**ΔDifference in mean change (95% CI)**
**HbA1c (%)**					
Vildagliptin	8.75 ± 1.15	7.41 ± 1.43	-1.34 (-2.03, -0.64)**		
Saxagliptin	8.86 ± 1.13	7.65 ± 1.41	-1.21 (-1.91, -0.51)**	0.13 (-0.66, 0.40)	
Sitagliptin	8.54 ± 1.19	7.47 ± 1.42	-1.07 (-1.64, -0.50)**	0.27 (-0.80, 0.26)	0.14 (-0.67, 0.39)
**FBG (mmol/L)**					
Vildagliptin	8.79 ± 1.80	6.35 ± 1.57	-2.44 (-3.01, -1.87)**		
Saxagliptin	8.36 ± 2.04	6.53 ± 1.92	-1.83 (-2.13, -1.53)**	0.61 (0.30, 0.92)##	
Sitagliptin	8.22 ± 1.77	6.73 ± 1.69	-1.49 (-1.69, -1.29)**	0.95 (0.64, 1.26)##	0.34 (0.03, 0.65)Δ
**P2BG (mmol/L)**					
Vildagliptin	11.98 ± 2.46	8.27 ± 2.28	-3.71 (-4.16, -3.26)**		
Saxagliptin	11.77 ± 3.07	8.36 ± 2.70	-3.41 (-4.33, -2.49)**	0.30 (-0.32, 0.92)	
Sitagliptin	10.98 ± 2.93	7.82 ± 2.58	-3.16 (-4.03, -2.29)**	0.55 (-0.07, 1.17)	-0.25 (-0.37, 0.87)

63 subjects in Vildagliptin-added group, 66 subjects in Saxagliptin-added group and 61 subjects in Sitagliptin-added group were analyzed; ^★^Difference in mean change calculated as Saxagliptin minus Vildagliptin or Sitagliptin minus Vildagliptin; ^Δ^Difference in mean change calculated as Sitagliptin minus Saxagliptin. CI: confidence interval; **p < 0.01 for mean change from baseline in Vildagliptin, Saxagliptin and Sitagliptin; ^##^p < 0.01 for the between-treatment difference from Vildagliptin; ^Δ^p < 0.05, ^ΔΔ^p < 0.01 for the between-treatment difference from Saxagliptin.

The patients achieving target HbA1c of less than 6.5% were 32% (21 patients) of saxagliptin-added group, 36% (23 patients) of vildagliptin-added group, and 25% (15 patients) of sitagliptin-added group, without significant differences among the three groups (p = 0.14). Those with HbA1c less than 7.0% were 59% (39 patients) of saxagliptin-added group, 65% (41 patients) of vildagliptin-added group, and 59% (36 patients) of sitagliptin-added group, with no significant differences among the three groups (p = 0.51).

### Adverse events

There were no deaths during the study. Serious adverse events, and adverse events (AEs) caused dose interruptions or dose changes were almost not happened, as well as, it is comparable between groups. Mild hypoglycemia was occurred in all groups (6% of saxagliptin-added group vs. 2% of vildagliptin-added group and 3% of sitagliptin-added group) (Table 3).

**Table 3:** Adverse events

**Adverse event**	**Saxagliptin n = 68**	**Vildagliptin n = 63**	**Sitagliptin n = 63**	**P value**
Edema	1(2%)	0(0%)	1(2%)	0.60
Headache	0(0%)	1(2%)	1(2%)	0.58
Elevated liver enzymes	2(3%)	3(5%)	2(3%)	0.86
Symptomatic hypoglycemia	4(6%)	1(2%)	2(3%)	0.41
Abdominal discomfort	3(4%)	5(8%)	3(5%)	0.64
Diarrhea	3(4%)	1(2%)	3(5%)	0.57
Chest discomfort & dyspnea	2(3%)	2(3%)	2(3%)	0.99
Nasosinusitis	5(7%)	0(0%)	6(10%)	0.053
Total	20(29%)	13(21%)	20(32%)	

Data are presented as number (%). *χ*^2^ test and Fisher’s exact test were used for comparison of differences in the three groups.

Other adverse events, including diarrhea, headache, edema, abdominal discomfort, elevated liver enzymes, nasosinusitis and chest discomfort or dyspnea were similarly happened among the three study populations. During 24-week study, one patient in saxagliptin-added group withdrawn from the study, who was discontinued for peripheral edema with unknown reasons.

## Discussion

The current study evaluated the efficacy and safety of adding saxagliptin, vildaglitpin or sitagliptin as add-on therapy to dual combination of metformin and other oral hypoglycemic agents (glimepiride, acarbose and pioglitazone) of patients with inadequately glycemic control. Adding treatment with saxagliptin, vildagliptin, and sitagliptin all resulted in significant improvements in glycemic control. There was no significant difference among the three treatment groups of HbA1c changes and P2hBG changes, as well as the proportion of achieving target HbA1c levels. Nevertheless, it was demonstrated that saxagliptin was superior to sitagliptin, while, inferior to vildagliptin in terms of changes in FBG after 24-week treatment. The incidence of adverse events with three DPP-4 inhibitors was similar, and all of them were well tolerated.

This study showed no significant reductions in HbA1c from baseline to week 24 among the three DPP-4 inhibitors-added groups, decreasing by 1.1 ~ 1.3%. Besides, about 60% of the study participants reached a target HbA1c of less than 7.0%, but the rest of the study participants remained in poorly glycemic control. And only about 30% of the subjects reached a target HbA1c less than 6.5%. While, previous placebo-controlled trials showed that the DPP-4 inhibitors could decrease HbA1c by about 0.6 ~ 0.9% [29-31]. When added to metformin, sulphonylureas or alpha-glucosidase inhibitors, DPP-4 inhibitors could reduce HbA1c by about 0.6 ~ 1.1% [32]. Moreover, adding DPP-4 inhibitors to ongoing metformin allowed approximately 30–50% patients to achieve HbA1c levels < 7.0% [33-36]. The differences between our results and previous studies might be influenced by different racial background of study populations, baseline HbA1c levels, BMI, types and dosages of background drugs and prescribed doses of the study drugs. Most of Previous reports were performed in western countries, a majority of participants are Caucasian, whose baseline HbA1c levels were 8.1-10%, and BMIs were 26 ~ 30 kg/m2 [33-38]. Whereas the patients of our study are Orientals, who had HbA1c levels of 7.5-10%, and BMIs of 22.5-30 kg/m2. Such difference might be attributed to the subjects in our study having relatively mild hyperglycemia and lower BMIs, as well as, maybe Orientals are more sensitive to DPP-4 inhibitors. Several clinical trials also showed similar HbA1c reductions (1.1 - 1.4%) with our results in Asian countries [27,39].

As indicated in our study, vildagliptin showed the greatest reductions of FBG, while, sitagliptin showed the smallest reductions. This might be explained by the structure of the three DPP-4 inhibitors. Saxagliptin and vildagliptin are cyanopyrrolidines that form a covalent bond with the active site serine [40], which may result in more robust inhibition to DPP-4 and greater potency of lowering blood glucose level than sitagliptin with nonvalent bond with DPP-4. And it is might be relational that the usage of vildagliptin was twice daily, while, saxagliptin and sitagliptin was once daily. A previous study showed that vildagliptin induced better circadian glycaemic control than sitagliptin with a significant decrease on overall hyperglycemia, mainly driven by reduction on basal hyperglycaemia [41]. Several Asian clinical trials demonstrated that vildagliptin 50 mg twice daily was associated with significantly greater HbA1c reduction than sitagliptin 100 mg once daily in patients of mean HbA1c ranged from 7.4% to 7.8% [42-44].

The reported numbers of hypoglycaemic events in studies with vildagliptin and sitagliptin are very low. A pooled analysis for sitagliptin in monotherapy showed that the incidence of hypoglycaemia was 1.2% after treatment with sitagliptin at 100 mg daily versus 0.9% in the placebo group [20]. Also as add-on therapy to metformin, DPP-4 inhibitiors showed a low degree of hypoglycaemia. Thus, when compared to sulphonylurea as add-on to metformin, sitagliptin showed substantially lower risk of hypoglycaemia (5%) than glipizide (32%) [45]. When combined with sulphonylurea, DPP-4 inhibitions showed increased risk of hypoglycaemia. Thus, when sitagliptin was added to ongoing glimepiride, there was an increased incidence of hypoglycaemia (12% vs. 2% in the group given glimepiride alone) [38]. Furthermore, when vildagliptin was added to glimepiride, a slight increase in the incidence of hypoglycaemia was reported (1.2% vs. 0.6% during treatment with glimepiride alone) [37]. Overall, these studies show a low risk of hypoglycaemia during treatment with DPP-4 inhibition.

In many previous studies, the overall prevalence of adverse events was similar to that seen in placebo groups both in monotherapy and in combination therapy with metformin, sulphonylurea or alpha-glucosidase inhibitors. Nevertheless, in some studies, adverse events such as upper respiratory tract infection, nasopharyngitis and headache were reported, although the association of these adverse events with the compounds has not been established. So far, vildagliptin and sitagliptin have now been examined in a large number of subjects and shown to be tolerable and safe, both in short-term studies and in studies up to 1 year of duration [27,37-39,46]. Similarly, saxagliptin is also tolerable and safe [29,47], although experience with this compound is more limited. The main adverse event with metformin was diarrhea, with glimepiride was hypoglycemia and weight gain, with pioglitazone was edema due to fluid retention and weight gain. However, the symptoms were transient in the majority of the study participants and the causal relationship between the study drugs and symptoms is uncertain.

## Limitation and conclusion

There were still several limitations in our study. Firstly, 24 weeks is too short a study duration to evaluate long-term glycemic control, weight loss, and β-cell preservation. Secondly, patients were enrolled based on specific criteria and were followed according to the study schedule, which may not reflect the real clinical use. Thirdly, as the patients were evaluated at outpatient, no specific compliance data were collected. Moreover, our study is small and had an open-label design, which are both limiting factors for the generalization of the data, the limitation should be considered.

The current study demonstrated that the efficacy and tolerability of saxagliptin, vildagliptin, and sitagliptin are similar, with no significant differences, when used to treat type 2 diabetic patients with inadequate blood glucose control by dual combination of metformin and another traditional oral hypoglycemic agent (glimepiride, acarbose, or pioglitazone). But vildagliptin showed grater FBG reduction than sitagliptin. This trial is the first randomized controlled trial to evaluate the efficacy of commonly-used antidiabetic agents in Chinese type 2 diabetic patients. Specific characteristics of the study drugs should be considered when choosing an appropriate agent. To use these results as valuable information for selecting an oral hypoglycemic agent, more detailed subgroup analyses and further investigation would be recommended.

## Competing interests

The authors declare that they have no competing interests.

## Authors’ contributions

DMY and CJL conceived the study, analyzed data and wrote the manuscript. CJL XJL and LB acquired and analyzed data, QY, QMZ and PY wrote the manuscript. All authors read and approved the final manuscript.
